# An exercise intervention in children and young adults with McArdle disease: feasibility, acceptability, and clinical outcomes

**DOI:** 10.1186/s13023-026-04222-8

**Published:** 2026-01-31

**Authors:** Kiera Batten, Nancy Van Doorn, Christopher McManus, Ben Jones, David Simar, Carolyn Broderick, Kaustuv Bhattacharya

**Affiliations:** 1 Department of Nutrition and Dietetics, The Children’s Hospital at Westmead, Sydney, Australia; 2 Genetic Metabolic Disorders Service, The Children’s Hospital at Westmead, Sydney, Australia; 3 Faculty of Medicine and Health, School of Health Sciences, UNSW, Sydney, Australia; 4 Children’s Hospital Institute of Sports Medicine, The Children’s Hospital at Westmead, Sydney, Australia; 5 University of Essex, School of Sport, Rehabilitation and Exercise Sciences, Colchester, UK; 6 Faculty of Medicine and Health, School of Clinical Medicine, UNSW, Sydney, Australia

**Keywords:** Paediatric, GSD V, CPET, Remote, Safety, NIRs

## Abstract

**Introduction:**

Patients with McArdle disease have reduced exercise capacity. Structured exercise programs in adults with McArdle disease can improve aerobic capacity and strength. However, structured exercise has not been evaluated in younger populations. Our aim was to determine the safety, feasibility, and acceptability of an exercise intervention in children and young adults with McArdle disease.

**Methods:**

Children and young adults aged 5–30 years with McArdle disease were recruited through metabolic clinics in New South Wales, Australia, to complete a remote, supervised 12-week exercise intervention. Pre and/or post intervention, participants completed a treadmill cardiopulmonary exercise test (CPET), strength testing, habitual physical activity monitoring, a quality-of-life questionnaire, acceptability questionnaire, muscular Near Infrared Spectroscopy (NIRS), and blood samples.

**Results:**

Five out of 10 eligible participants, with a median age of 17 years (range 13–29), were enrolled. Four met the feasibility target of 70% completed exercise sessions. Four episodes of mild rhabdomyolysis were reported during the study, but no participant required hospital admission. All participants reported they would participate in similar programs in the future. No significant changes were found in aerobic capacity, strength, habitual physical activity levels or quality of life. Trends were observed for lower perceived pain during CPET, and improved leg press. NIRS indicated a possible trend for improved muscle oxygen utilisation.

**Conclusion:**

A 12-week remotely delivered exercise intervention was found to be feasible, safe, and acceptable to children and young adults with McArdle disease. Although improvements to aerobic capacity and strength were not elicited, individual clinical benefits may have occurred.

**One sentence take home message:**

An exercise intervention in children and young adults with McArdle disease is feasible, safe, and acceptable, and may elicit individual clinical benefits.

**Supplementary Information:**

The online version contains supplementary material available at 10.1186/s13023-026-04222-8.

## Introduction

McArdle disease (Glycogen Storage Disease type V) (OMIM #232600), arises due to deficiency of skeletal muscle glycogen phosphorylase (EC 2.4.1.1) [1]. Deficiency of this enzyme prevents the conversion of muscle glycogen to glucose-1-phosphate, reducing muscle energy production due to the absence of anaerobic glycolysis [1]. This results in muscle pain, cramping, weakness, and stiffness, during or after physical activity [1–11]. Rhabdomyolysis can also occur after exercise in McArdle disease, manifesting as elevated creatine kinase (CK) in the bloodstream, as well as myoglobinuria, deranged liver function tests, and occasionally impaired kidney function [10, 12]. McArdle disease also features a pathognomonic phenomenon, ‘the second wind.’ This phenomenon is characterised by an exaggerated increase in heart rate, perceived exertion, and muscle pain at the beginning of exercise, followed by normalisation around the 7th minute of exercise [10, 13–17]. Symptom onset can be directly attributed to the glycogen phosphorylase enzyme deficiency, with subsequent improvement due to utilisation of alternative substrates [13, 14, 18]. 

Multiple small cohort and case studies have reported that patients with McArdle disease typically exhibit a peak oxygen uptake (VO_2_peak) of ¬50% of controls, predicted values, or normative data [6, 12, 16, 19–34]. This reduced aerobic fitness is likely to have a significant impact on an individual’s ability to undertake exercise, including activities of daily living (ADLs) [18, 35–37]. 

Clinical practice guidelines recommend adults with McArdle disease participate in regular exercise, to improve VO_2_peak, muscle power, functional capacity, and quality of life, as well as reduce clinical severity [10, 18, 19, 21, 38–40]. Only two case reports have been published on exercise interventions in paediatric patients [41, 42]. Thus, no firm conclusions on training outcomes, feasibility or safety of these programs can be extrapolated.

The primary aim of our study was to determine the feasibility, acceptability, and safety of a structured, supervised exercise program in young people with McArdle disease. The secondary aims were to explore the impact of the exercise intervention on aerobic capacity, strength, QoL, and habitual physical activity levels.

## Methods

### Recruitment

Potential participants were identified through the Sydney Children’s Hospital Network (SCHN) and Westmead Adult metabolic clinics in NSW, Australia. Inclusion criteria comprised a confirmed diagnosis of McArdle disease, and aged 5–30 years old. The study was approved by the SCHN Human Research Ethics Committee (2023/ETH01965). Potential participants and/or carers were emailed study information, followed up by phone call, completed informed written consent, and recruited.

## Study design

**Fig. 1:**
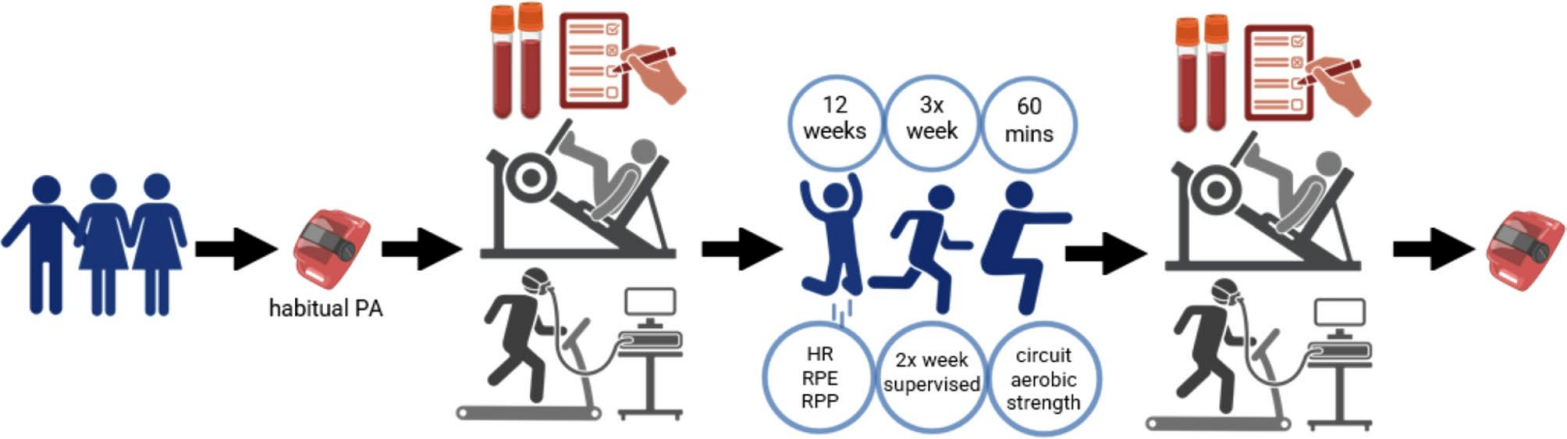
Study design diagram. Created with BioRender.com

Each participant completed treadmill and strength testing twice, at baseline (visit 1) and after the 12-week exercise intervention (visit 2) (Fig. 1). A physical activity monitor (Actigraph wGT3X-BT, Actigraph, USA) was mailed to each participant before visit 1, and given back at visit 2, with instructions to wear continuously for 7 days before visit 1 and after visit 2. Participants were also provided a resistance band and wrist heart rate (HR) monitor (FitBit Inspire 3, Google, USA), used during individual exercise sessions. Participants were emailed calendar invites (Microsoft Teams) for supervised exercise sessions.

## Pre-exercise testing

Participants consumed the same breakfast a minimum two hours before each visit. Upon arrival, body mass and height were measured and body mass index (BMI) calculated. Participants (and their carers if < 18 years old) were asked to complete the Patient-Reported Outcomes Measurement Information System (PROMIS) Global Health QoL questionnaire (visit 1 and 2) and an acceptability questionnaire (visit 2 only, see supplementary data Fig. 5) [43]. Blood pressure and heart rate were measured at rest prior to, and at completion of exercise testing (Dinamap Pro 100V2, GE Healthcare, USA).

## Exercise testing

A Cardiopulmonary Exercise Test (CPET) was conducted on a treadmill (Medisoft Model 870 A, MGC Diagnostics, USA), with continuous HR monitoring (H10, Polar Electro 2025, USA) [36]. Respiratory gases were collected using a mask (7450 V2, Hans Rudolph Inc, USA) and analysed breath by breath (Ultima System Cardio2 model 800840-021, MGC Diagnostics, USA). Muscle oxygenation across the right lateral gastrocnemius was continuously measured using Near Infrared Spectroscopy (NIRS) (PortaMon, Artinis Medical Systems, BV, The Netherlands) [44]. The exercise protocol was undertaken as: 10 min warm up at 2.4 km/hr and 0% incline to evoke second wind, followed by the STEEP protocol, then a three minute warm-down at 2.4 km/hr and 0% incline, before CPET termination [45]. Ratings of perceived exertion (RPE) and perceived pain (RPP) were taken at the end of every minute (modified Borg scale 1–10) [46]. Criteria for maximal effort included VO_2_ plateau despite workload increase, HRpeak ≥ 95% predicted max for < 18yo or ≥ 85% for ≥ 18yo, Respiratory Exchange Ratio (RER) peak > 1.00 for < 18yo or ≥ 1.10 for > 18yo; or RPE ≥ 9 [47–51].

Strength testing comprised a long jump, leg press, lat-pulldown, and bicep curl. The lower body was presumed to be in second wind immediately following CPET. An upper body warm-up was conducted via unweighted arm movements, and low-weight repetitions. Participants were instructed to complete 5–6 repetitions maximum (RM) on each strength exercise with the highest weight possible to achieve this number of repetitions. The long jump was taken as the best of two tries.

## Pathology

Venous blood samples were taken before, and within two hours after exercise testing, at both visits. Analytes tested included CK, aspartate transaminase (AST), alanine transaminase (ALT).

### Exercise intervention

Exercise sessions were scheduled three times a week for an hour, for 12 weeks. Sessions were not rescheduled if missed. Exercise commenced with walking until second wind, followed by a warm-up set of all resistance exercises. A circuit-based format then ensued, targeting 3 sets of 5–6 strength exercises, and 1–4 aerobic exercises. Strength examples included squats, calf raises, push-ups, crunches, biceps curls. Aerobic exercise examples included high knees, side shuffles, jogging, elliptical and bike machines. At the end of each set, the participant reported their HR, RPE and RPP [46]. Target HR was 50–75% of predicted maximum (208-(0.7*age)); target RPE was 5–7; and RPP 0–1 [18, 38, 52, 53]. 

## Supervision

Participants or carers were contacted the day after exercise testing, to report adverse events. If rhabdomyolysis was established via CK rise and concurrent myopathic symptoms, exercise sessions were postponed for two weeks. Weekly exercise prescription was individually revised and overseen by an accredited exercise physiologist. Two of three exercise sessions were supervised remotely by an exercise physiology student or an exercise scientist. If an adverse event occurred, a study investigator reviewed the patient via phone, and investigations were requested if rhabdomyolysis was suspected.

## Data processing

NIRS raw data was extracted from OxySoft Version 3.2.72 and cleaned in Microsoft Excel. The data was pre-processed to remove high-frequency noise and outlier artifacts. A 10th -order zero-phase Butterworth filter (cutoff = 0.1 Hz) was applied using MATLAB (*filtfilt* function), to ensure the signal phase was preserved [54, 55]. Tissue Saturation Index (TSI) was expressed as a percentage and determined as: [O2Hb]/([O2Hb] + [HHb]) x 100. It was calculated using the spatially resolved spectroscopy method. Actigraph data was analysed with ActiLife v6.13.3, using 60s epochs, and the Choi non-wear and Freedson Adult 1998 cut-point algorithms to calculate moderate to vigorous physical activity (MVPA) [56–58]. An ‘invalid’ day was determined as more than 14 h of non-wear time within 24 h; and an ‘invalid’ week more than 3 ‘invalid’ days [58]. 

### Statistical analysis

Statistical analysis was conducted using SPSS Statistics Version 27 (IBM, USA). Changes to outcomes pre and post intervention were analysed using Related-Samples Wilcoxon Signed Rank Test with a significance level of 0.05. Graphs were produced via Prism V10.4.1 (Graphpad, USA). Study feasibility was determined using opt-in and retention rates, proportion of individual sessions completed, and number of technological difficulties. Safety was determined using number of serious adverse events including hospital admissions. Due to small sample size and age of enrolled participants, some results are presented and discussed individually, rather than stratified to ‘children’ and ‘young adults.’

## Results

### Feasibility

**Fig. 2:**
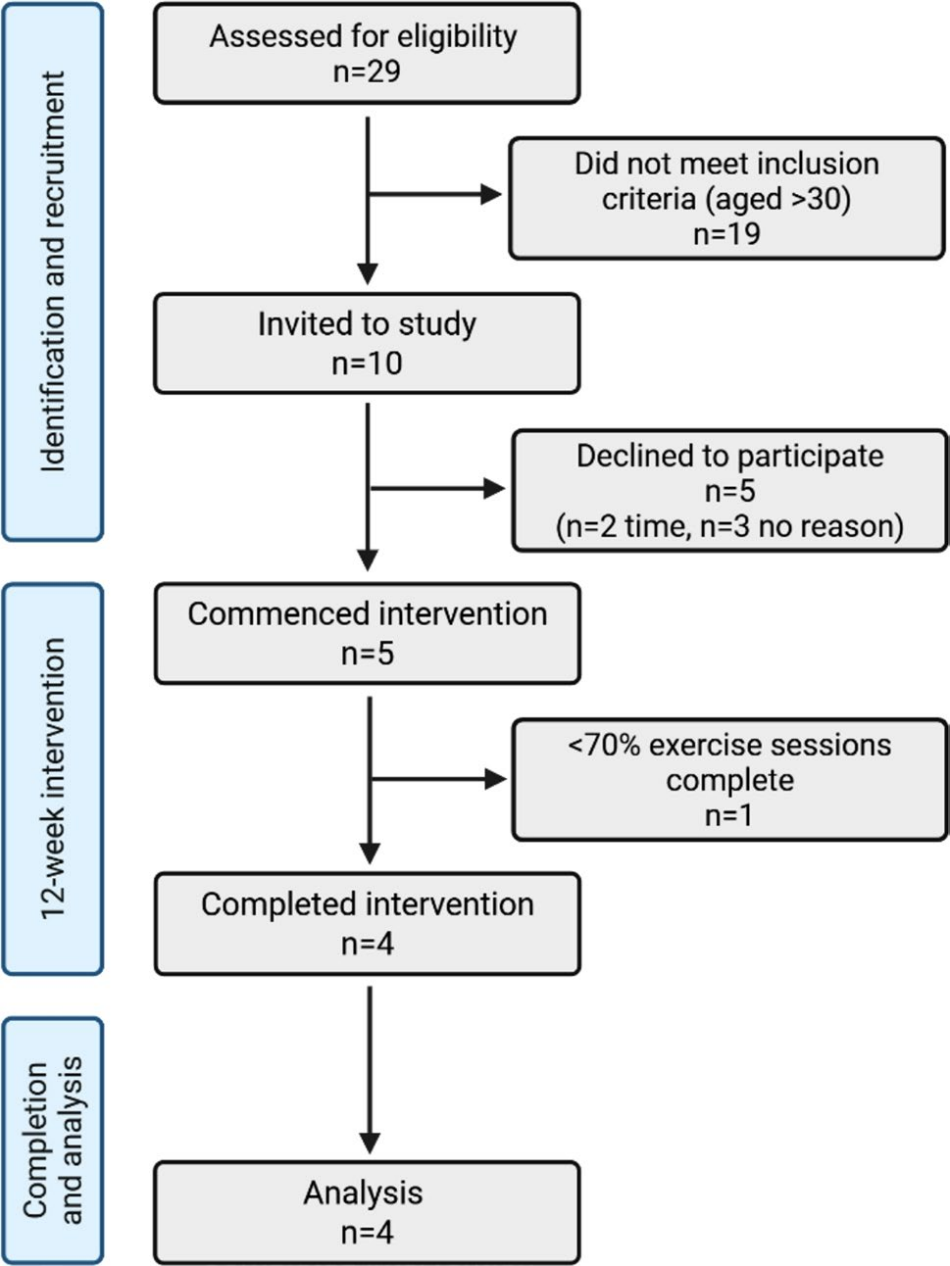
Consort flow diagram for participant study entry. Created with BioRender.com

Of ten eligible, invited participants, five (median age 17 years) were consented and recruited to the study (Fig. 2). Participants demographics can be found in Table 1. Participants completed a median of 29/36 (81%) exercise sessions, with 4/5 participants meeting individual target feasibility of ≥ 70% sessions completed [59, 60]. Reasons for missing sessions included conflicting priorities (work, family, or sport), illness, forgetting, and intentional delay following rhabdomyolysis.

**Table 1:** Participant demographics

Participant	Age	Sex	Age at symptom onset	Age at diagnosis	Reported episodes rhabdomyolysis in previous 12 months	Hospitalisations due to rhabdomyolysis in previous 12 months	Allele 1	Allele 2	Weight (kg)	Height (cm)	BMI (kg/m^2^) CDC category [61]
1	17	M	5	8	1 (medical file)	1 (medical file)	PYGM: c.148 C > T (causing p.Arg50*)	PYGM: c.613G > A (causing pGly205Ser)	62.2	171.8	21.1 HWR
2	17	M	7	8	3 (medical file)	3 (medical file)	PYGM: c.148 C > T (causing p.Arg50*)	PYGM: c.613G > A (causing pGly205Ser)	78.1	179.3	24.3 HWR
3	13	F	5	7	0	0	PYGM: c.148 C > T (causing p.Arg50*)	c.253_254insTA (causing p.Tyr85fs*5)	51.7	166.8	18.6 HWR
4	26	F	5	19	0 (medical file)	0	PYGM: c.148 C > T (causing p.Arg50*)	c.253_254insTA (causing p.Tyr85fs*5)	68.7	171	23.5 HWR
5	29	M	17	18	0 (medical file) 4 (self-reported)	0	PYGM: c.148 C > T (causing p.(Arg50*)	PYGM: c.613G > A (causing p.(Gly205Ser)	118.6	187	33.9 Above HWR

F=female; M=male; BMI=body mass index; fs=frame shift; HWR = healthy weight range; PYGM = gene that encodes muscle glycogen phosphorylase. CDC = centre for disease control and prevention. Weight, Height, BMI at visit 1

There were 21 technological difficulties reported during the intervention, of 126 completed sessions. These included forgetting HR monitor, HR monitor not charged or reading HR; connection difficulties with Microsoft Teams, incorrect video link used by participants, and poor WIFI/mobile signal. These were not a barrier to participation in the study.

Of the 2701 h reported throughout the exercise intervention, 2299 (85%) met target range of 50–75% predicted maximum HR, indicating the exercise intensity was consistent with what was proposed in our study design [18, 38].

### Safety

Nine adverse events were reported during the study. These included four episodes of rhabdomyolysis, three of which occurred after exercise testing (Table 2). The remainder included temporary muscle soreness, and one episode of emesis. No participant was hospitalised during the study. No difference was found between baseline CK at visit 1 and visit 2 (p-value 0.27). CK was elevated post exercise testing compared to baseline (p-value < 0.01), with AST and ALT mildly elevated across most time points (See supplementary data Tables 5, 6 and 7).

**Table 2:** Episodes of rhabdomyolysis during the study. Baseline CK taken as the pre-exercise test CK at either visit 1 or 2, depending on timing of rhabdomyolysis. U/L = units per litre

Participant	Suspected Precipitant	Creatine Kinase (CK)			Reported Symptoms
		Baseline (U/L)	24–48 h (U/L)	Reference range (U/L)	
4	Testing Visit 1 Lat pulldown	485	8111 (24 h)	45–250	‘Flu-like symptoms,’ significant upper body pain, generalised whole-body fatigue and weakness, peripheral tingling.
5	Testing Visit 1 Lat pulldown	634	Medical PI unable to contact for initial 48 h	45–250	Swollen, stiff and painful back. ‘Mild’ urine colour changes for 12 h. Weak, loss of stamina for two days.
4	Unsupervised session week 12 Glute bridges	485	7242 (24 h) 4830 (48 h)	45–250	Initially isolated quadriceps pain, subsequent all over body pain and fatigue. Mild peripheral tingling.
4	Testing Visit 2 Treadmill	1098	2694 (48 h)	45–250	Pain in upper back and diaphragm, especially upon deep inhalation. ‘Flu-like symptoms,’ peripheral tingling, general lethargy.

### Acceptability

80% of participants reported they enjoyed the exercise intervention “very much” and 100% “would participate in a similar program in future.” All participants felt the duration, intensity and frequency were “just right.” All participants also felt it improved their ability to do other exercise. Individual participant feedback included: “[it was] good for learning to complete regular, effective and safe exercise in the future;” “[the] difficulty level was reliably challenging but not uncomfortable;” “[I have] new-found confidence;” “…a controlled, supportive, and educational department;” and “…it was fun.”

### Aerobic fitness

All participants achieved second wind during the warm-up phase of CPET, as determined by reduction in HR, RPE and/or RPP. VO_2_peak was low, on average 41% of predictive norms (Table 3). Muscular pain was stated as the reason for CPET termination in four participants; and effort and central fatigue in one. HR approached predicted maximum in three participants at both visits. RER reached ≥ 1.0 in three participants on one exercise test each.

**Table 3:**
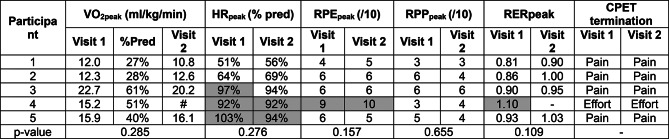
Cardiopulmonary exercise test (CPET) outcomes at visit 1 and visit 2. VO_2_peak = peak oxygen uptake; HRpeak = peak heart rate; %pred = percent of predicted (VO_2_peak compared to norms; HRpeak compared to Taneka formula); RPE = rating of perceived effort (modified Borg scale); RPP = rating of perceived pain (modified Borg scale); RER = respiratory exchange ratio; #=equipment failure. Greyed out cells meeting ‘maximal effort’ criteria

The participant who did not complete the minimum number of exercise intervention sessions was excluded from pre/post analysis. No significant change was found in aerobic capacity before and after the exercise intervention (p-value > 0.05) (Table 3). There was no difference found in total time of exercise during CPET between visit 1 and 2, number of full stages completed, number of treadmill step-offs, or magnitude of heart rate change during the second wind. Only participant 4 appeared to reach ventilatory threshold (VT), calculated via the ventilatory equivalents method, which occurred at 97% of VO_2_peak [48, 62]. (See supplementary data Tables 1, 2, 3 and 4; Fig. 1).

**Fig. 3:**
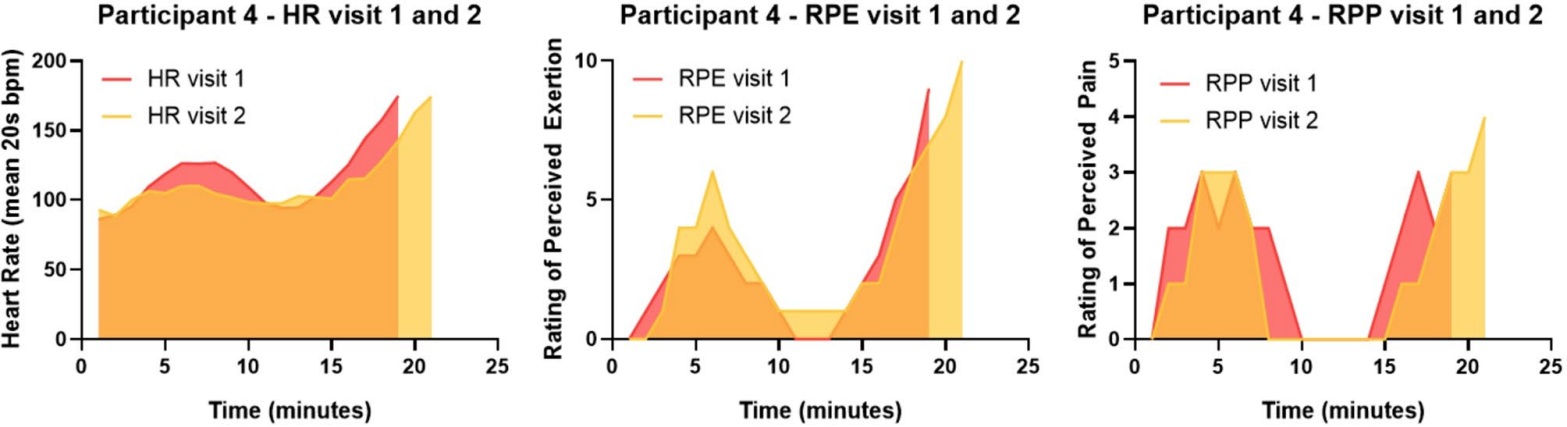
Participant 4 heart rate, perceived exertion (RPE) and perceived pain (RPP) during CPET at visit 1 and 2

We identified a trend for lower total RPP score in response to CPET at visit 2 compared to visit 1 (p-value 0.07) (see supplementary data Table 8). Participant 4 also appeared to have a reduced HR for equivalent stages and workloads, at visit 2 compared to visit 1 despite similar RPE (Fig. 3). This HR trend was not observed in other participants, possibly due to treadmill step-offs (participant 1 and 2) and strenuous exercise undertaken in the prior 48 h to visit 2 (participant 3).

### Muscle strength

**Fig. 4:**
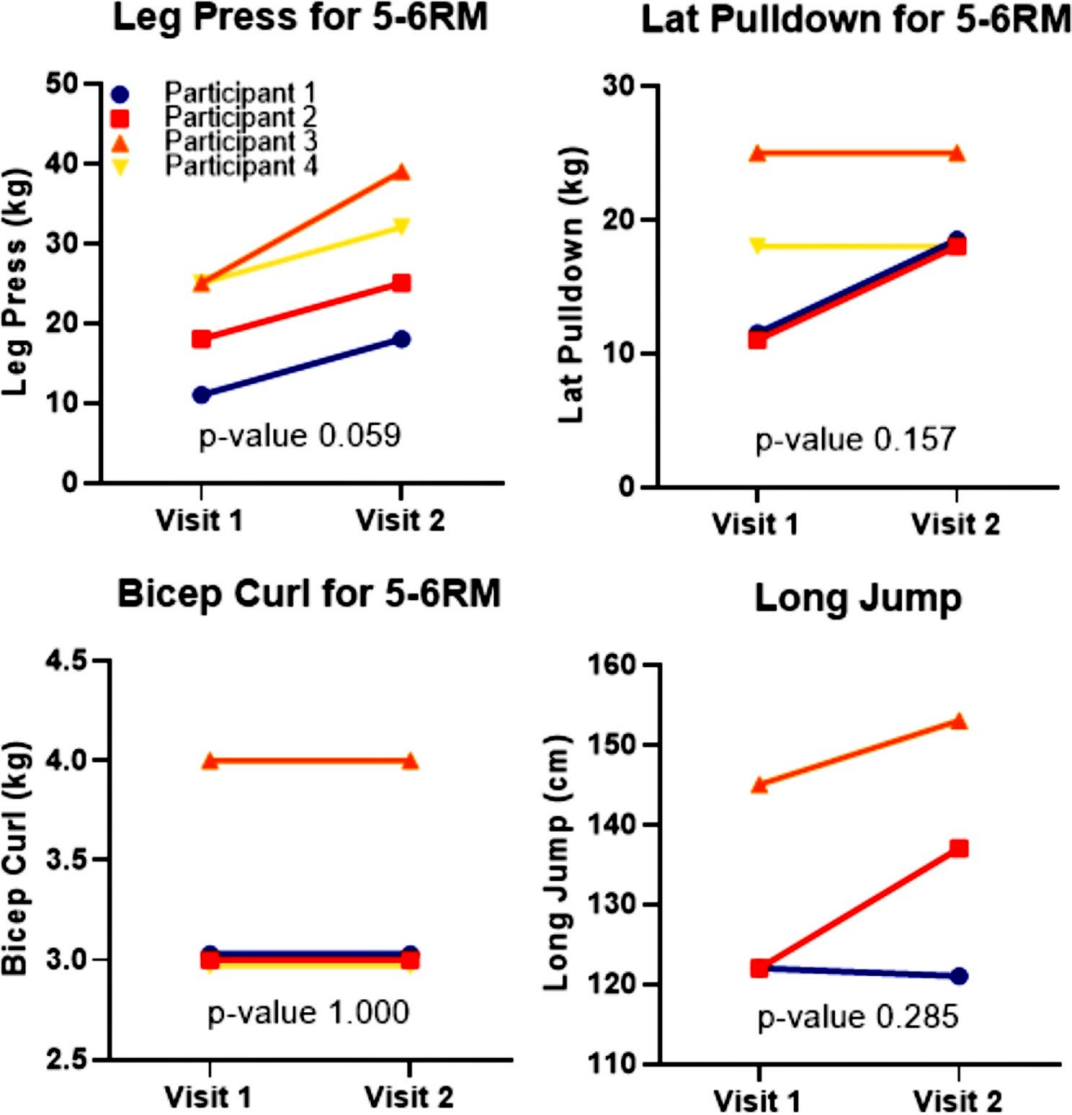
Strength testing at visit 1 and visit 2. Weight lifted was for 5–6 repetitions maximum (RM). Participant 4 had a pre-existing knee injury and thus did not partake in long jump testing

On observation, two participants increased in both completed strength sets (2 to 5) and repetitions (2–4 to 4–6) throughout the intervention. All participants progressed in complexity of strength exercises. There was no significant change found on any strength exercise, though leg press trended towards an improvement with a 47% increase in weight lifted for 5-6RM (p-value 0.06) (Fig. 4).

### Muscle oxygenation

Three of four participants completed NIRS assessment during CPET at visit 1 and visit 2. Results suggested a post-intervention trend towards reduced TSI % and increased HHb during exercise, interpreted as improved oxygen utilisation in the muscle; (mean change − 2.3%, p-value 0.11, and 1.5 μm, p-value 0.11, respectively). Two participants also demonstrated a trend for steeper TSI % slope (rate of change) during the CPET recovery period, suggesting improved reoxygenation post exercise. (See supplementary data Figs. 2, 3 and 4)

### Quality of life

**Fig. 5:**
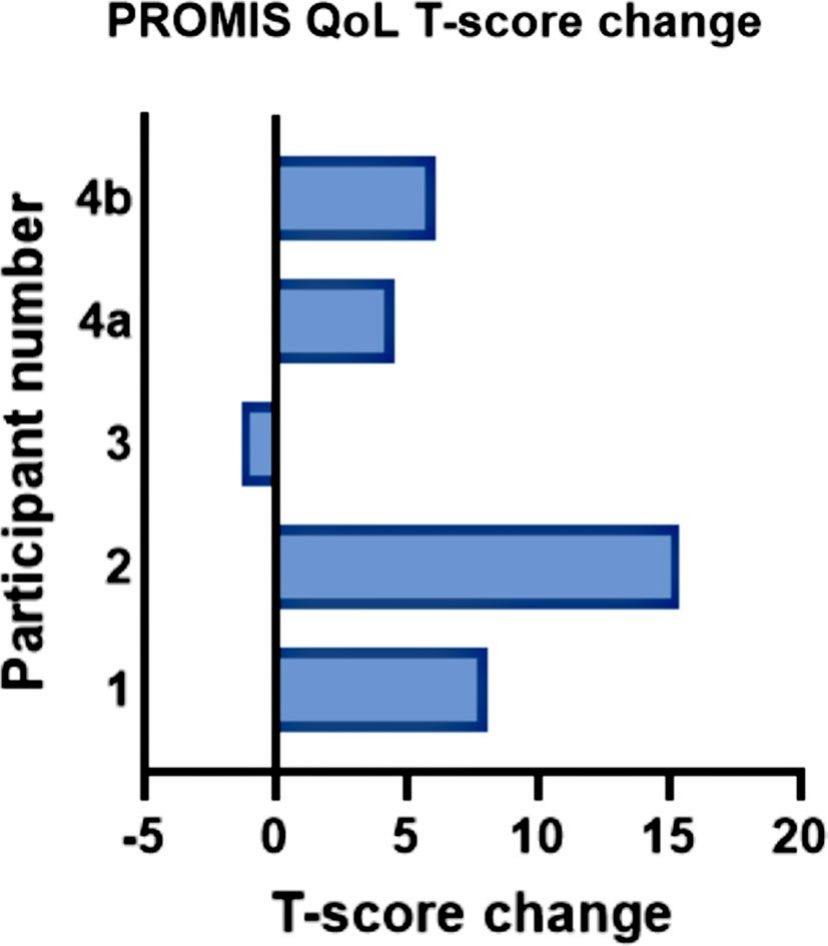
Change to PROMIS global health T-score pre and post intervention. Participants 1–3: Global Health (combined). Participant 4: 4a = global mental health; 4b = global physical health. NB PROMIS Global QoL output reported as single T-score in paediatric questionnaire but split into ‘physical’ and ‘mental’ domains for adult questionnaire

A trend was observed for improved global QoL between pre and post intervention as determined by change in T-score (p-value 0.08, Fig. 5).

### Physical activity

Of the three participants with adequate Actigraph wear time, there was no difference in minutes of MVPA pre and post intervention (p-value 0.59), nor steps per day (p-value 0.11) [58, 63]. These three participants only met 16–69% of World Health Organisation (WHO) recommended MVPA weekly minutes [64–66]. Reasons for low Actigraph wear time included irritation against the skin, unrelated illness, forgetting to reattach after shower or sleep, and inability to wear under sporting gear.

## Discussion

Our study evaluated the impact of a 12-week exercise intervention in young people with McArdle disease, finding the intervention to be feasible, safe, and acceptable. Although our participants did not demonstrate any significant changes to aerobic capacity, strength, QoL or habitual MVPA, individual clinical changes may have been elicited.

Compared to adult exercise studies in McArdle disease, our study had lower absolute participant numbers, despite a 50% opt-in rate [12, 40, 67–69]. However, our study had similar exercise adherence rates to adult McArdle studies of 74–100%, indicating that once enrolled, participants can maintain engagement [40, 69, 70]. 

None of the five existing adult studies reported rhabdomyolysis [12, 40, 67–69]. The mild rhabdomyolysis episode in our participant was likely due to inadvertent isometric contractions between reps, and miscommunication of prescribed weight. Despite this, program engagement and satisfaction were high from all participants.

No other exercise studies in McArdle disease have assessed intervention acceptability. Other remote-delivered paediatric exercise or rehabilitation programs in clinical populations have demonstrated lower or similar satisfaction levels to our study [60, 71, 72]. We believe individualised programs combined with regular supervision were key factors for a positive experience. Based on the established feasibility, acceptability, and safety of our intervention, similar programs could be confidently implemented in larger young McArdle cohorts.

Unlike adult studies, we found no group improvement in aerobic capacity in response to our exercise intervention [10, 19, 21, 39, 40]. However, individual benefits may be inferred by a trend for reduced RPP for the same CPET workload. While we utilised similar training principles to the existing exercise interventions, our training and CPET modalities were different. Most adult studies used cycle ergometry in both training and CPET, while we used a variety of aerobic training styles, with treadmill CPET [12, 40, 67, 68]. Choosing a CPET modality specific to the prescribed training is more likely to reflect exercise adaptations [73, 74]. 

VO_2_peak derived from maximal CPET can predict an individual’s capacity to undertake independent ADLs. Many adults with McArdle disease fall below the minimum VO_2_ threshold for independent living (15 ml/kg/min in women and 18 ml/kg/min in men) [21–34, 75–77]. Only two of our participants met these thresholds, and the other three fell below. However, all were able to maintain an independent life, including school, manual labour, or caring for dependents. This discrepancy may indicate that VO_2_peak value, or the response to exercise in the present study, might not accurately reflect the ability to undertake independent ADLs in our cohort. The calf, quadriceps and hamstring pain leading to CPET termination in most of our participants may infer these muscles had not achieved muscle-specific second wind for the incline-heavy STEEP protocol, despite a 10-minute warm up [18, 38, 78]. Myopathic symptoms limiting CPET have been reported previously in McArdle disease [8, 19, 25, 77]. 

Achieving adequate muscle-specific second wind poses a challenge in determining the most appropriate and specific treadmill CPET to utilise in McArdle disease, as maximal protocols typically involve increased slope, and/or speed [51]. Alternative protocols could consider a longer warm-up with incline or jogging, however this may lengthen CPET duration and increase fatigue prior to maximal effort [51, 79]. Cycle ergometry may be more tenable in replicating muscle movements across warm-up and maximal effort. However, young children typically have low familiarity with cycling, and difficulty maintaining constant cadence as experienced in our clinical exercise laboratory [49, 78]. We chose a walking protocol as a common, familiar and safe activity for young people. In the context of myopathic symptoms, and possible risk of rhabdomyolysis from maximal CPET, submaximal CPET or field tests may have more clinical utilitity with lower risk in McArdle disease, particularly for younger individuals [17, 76, 80]. A recent study established reference values for a modified 12 min walk test based on 103 adults with McArdle disease [19, 80]. This field test may be more clinically relevent in assessing change to aerobic and functional capacity compared to traditional CPET. However, normative values would need to be established in paediatric populations to support interpretation of the performance.

While clinical practice guidelines recommend regular exercise for McArdle disease, there are no optimal protocols specified to safely assess aerobic capacity and strength in this cohort [18, 38]. We reported one episode of rhabdomyolysis after CPET, and two after lat-pulldown testing. It is possible our strength warm-up protocol was insufficient, and/or participants may have over-extended in intensity, time, or isometric contractions between repetitions [38]. The arm crank ergometer recommended in clinical guidelines is not always available in research or fitness centres, and there is inadequate evidence to demonstrate its efficacy to warm-up all upper body muscles [38, 81, 82]. Only two studies have outlined strength-testing protocols in adults with McArdle disease, both using dynamic machines to determine concentric force and power for 1-3RM exercises [42, 69]. We could not measure force and power, thus chose a 5-6RM test as this reflects McArdle strength training recommendations, and is less demanding in intensity and form compared to 1RM [38, 83]. Based on our study, provided an adequate warmup is conducted, a 5-6RM test is safe and could be effective in assessing strength changes in McArdle disease.

NIRS is a non-invasive method increasingly used to monitor local muscle oxygenation dynamics including oxygen delivery and extraction, during exercise [26, 44]. Our NIRS results could indicate the exercise intervention improved local muscle oxygen extraction and utilisation during exercise, and enhanced reoxygenation post exercise. However, substantial variance between patients, and technical limitations including lack of correction for adipose tissue thickness, may have impacted signal quality [84]. Only two other studies have utilised NIRS in adults with McArdle, both describing reduced capacity for oxygen extraction during exercise, while one also established a positive relationship between peak muscle oxygenation and VO_2_peak [26, 85]. While our data suggests structured exercise may improve local muscle oxidative function in McArdle disease, further research with larger samples, standardised NIRS protocols, and comparative normative datasets is required.

In our study, none of the three participants improved their MVPA as a result of the intervention, nor did they meet the WHO activity guidelines [64–66]. However, MVPA may not be accurately captured during resistance training if the Actigraph is worn on the waist as was the case in our study [58, 86, 87]. Only two other studies have evaluated habitual physical activity in McArdle disease, with only 41 of 94 adults self-reporting they met WHO recommendations for MVPA [19, 21]. 

Three studies of a total 174 adults with McArdle disease have reported reduced QoL in this cohort, with physical functioning being the most affected of all domains [19, 88, 89]. Our participants had overall similar QoL scores to the general population. This disparity may be due to different QoL tools used (PedsQL, SF-36, PROMIS); few study participants, and different scoring outputs in PROMIS adult and paediatric questionnaires. No statistical change to global QoL occurred via the exercise intervention. However, a recent systematic review of 31 primarily adult studies suggested that a ‘minimal important change (MIC)’ exists with a 2–6-point difference in PROMIS T-score. Based on this MIC, some of our participants may have experienced individual improvements to global quality of life based on their T-score change and p-value trend [90]. 

### Limitations

As with all studies in rare disease, low participant numbers impacted our statistical analysis and thus conclusions drawn from the results should be considered with caution. These limitations were further compounded by data loss of one CPET, two participants’ Actigraph, and one participant’s NIRs. As explored above, the CPET treadmill protocol might have not been sensitive or specific enough for McArdle disease, particularly for assessing aerobic and functional capacity.

## Conclusion

In children and young adults with McArdle disease, a 12-week structured, supervised, remotely-delivered exercise intervention was found to be feasible, safe, and acceptable. The lack of statistical change to aerobic capacity, strength, QoL or MVPA might be explained by number of study participants and choice of exercise testing protocols, although participants may have experienced individual clinical benefits. The optimal testing protocols for aerobic capacity and strength are yet to be established in McArdle disease, especially in children. Additional studies with larger and younger cohorts will assist in assessing clinical, muscular, and exercise-related outcomes from exercise interventions in this population.

## Supplementary Information

Below is the link to the electronic supplementary material.

Supplementary Material 1: Supplementary material: Acceptability questionnaire, testing data, pathology, NIRs traces

## Data Availability

Data available on request from the authors.
