# Supplementary material for: An exercise intervention in children and young adults with McArdle disease: feasibility, acceptability, and clinical outcomes
Source: Orphanet J Rare Dis. 2026 Jan 31;21:78. doi: 10.1186/s13023-026-04222-8 (PMC12947363; doi:10.1186/s13023-026-04222-8)
Supplement: Supplementary file 1 — Supplementary Material 1: Supplementary material: Acceptability questionnaire, testing data, pathology, NIRs traces [file 13023_2026_4222_MOESM1_ESM.docx]

**Supplementary Data:**

**Table 1: Total treadmill time** (step off time not included)

| **Participant** | **Visit 1** | **Visit 2** | **Difference** |
| --- | --- | --- | --- |
| 1 | 802 | 993 | 191 |
| 2 | 962 | 1008 | 46 |
| 3 | 1228 | 1223 | -5 |
| 4 | 1130 | 1260 | 130 |
| 5 | 1128 | 1140 | 12 |
| p-value | 0.20 | | |

**Table 2: Completed treadmill stages**

| **Participant** | **Visit 1** | **Visit 2** | **Difference** |
| --- | --- | --- | --- |
| 1 | 4 | 6 | 2 |
| 2 | 7 | 7 | 0 |
| 3 | 10 | 10 | 0 |
| 4 | 8 | 11 | 3 |
| 5 | 8 | 9 | 1 |
| p-value | 0.18 | | |

**Table 3: Number of treadmill step-offs**

| **Participant** | **Visit 1** | **Visit 2** | **Difference** |
| --- | --- | --- | --- |
| 1 | 4 | 1 | 3 |
| 2 | 2 | 1 | 1 |
| 3 | 0 | 0 | 0 |
| 4 | 0 | 0 | 0 |
| 5 | 0 | 0 | 0 |
| p-value | 0.18 | | |

**Table 4: Magnitude of heart rate change during second wind**

| **Participant** | **Visit 1** | | | **Visit 2** | | |
| --- | --- | --- | --- | --- | --- | --- |
|  | **Baseline** | **Peak SW** | **Difference** | **Baseline** | **Peak SW** | **Difference** |
| 1 | 89 | 98 | 9 | 89 | 99 | 10 |
| 2 | 89 | 96 | 7 | 97 | 105 | 9 |
| 3 | 98 | 130 | 31 | 107 | 114 | 7 |
| 4 | 91 | 128 | 37 | 94 | 112 | 18 |
| 5 | 99 | 128 | 29 | 83 | 102 | 18 |
| p-value | 0.47 | | | | | |

**Table 5: Creatine Kinase**

| **Participant** | **Upper Limit** | **Visit 1** | | | **Visit 2** | | |
| --- | --- | --- | --- | --- | --- | --- | --- |
|  |  | **Pre CPET** | **Post CPET** | **Difference** | **Pre CPET** | **Post CPET** | **Difference** |
| 1 | 180 | 8667 | 9660 | 993 | 4944 | 6677 | 1733 |
| 2 | 180 | 5883 | 6512 | 629 | 4775 | 7970 | 3195 |
| 3 | 180 | 601 | 1357 | 756 | 569 | 643 | 74 |
| 4 | 250 | 485 | Haemolysed | - | 1098 | 1434 | 336 |
| 5 | 250 | 634 | 674 | 40 | 3396 | 3513 | 117 |
| p-value | | Baseline visit 1 vs visit 2: 0.273 \| Post CPET vs Pre CPET (visit 1 and 2): 0.008 | | | | | |

**Table 6: Aspartate transaminase (AST)**

| **Participant** | **Upper Limit** | **Visit 1** | | **Visit 2** | |
| --- | --- | --- | --- | --- | --- |
|  |  | **Pre CPET** | **Post CPET** | **Pre CPET** | **Post CPET** |
| 1 | 38 | 82 | 84 | 85 | 91 |
| 2 | 38 | 84 | 85 | 94 | 103 |
| 3 | 38 | 40 | 46 | 40 | 42 |
| 4 | 35 | 44 | Haemolysed | Haemolysed | 60 |
| 5 | 35 | Haemolysed | Haemolysed | 60 | 62 |

**Table 7: Alanine transaminase (ALT)**

| **Participant** | **Upper Limit** | **Visit 1** | | **Visit 2** | |
| --- | --- | --- | --- | --- | --- |
|  |  | **Pre CPET** | **Post CPET** | **Pre CPET** | **Post CPET** |
| 1 | 40 | 70 | 70 | 97 | 99 |
| 2 | 40 | 73 | 72 | 106 | 106 |
| 3 | 40 | 22 | 22 | 27 | 27 |
| 4 | 50 | 59 | Haemolysed | 52 | 51 |
| 5 | 50 | 99 | 98 | 74 | 73 |

**Table 8: Total RPP Scores for equivalent CPET stages at Visit 1 and Visit 2**

| **Participant** | **RPP Score Visit 1** | **RPP Score Visit 2** |
| --- | --- | --- |
| 1 | 27.0 | 10.0 |
| 2 | 58.5 | 24.0 |
| 3 | 42.0 | 19.5 |
| 4 | 28.0 | 20.0 |
| p-value | 0.07 | |

**Figure 1: Ventilatory Threshold Participant 4.**

**Figures 2-4: Example near infrared spectroscopy (NIRs) trace for relative deoxygenated haemoglobin (HHb) and % tissue saturated index (%TSI), Participant 2.**

**Figure 5: Acceptability Questionnaire:**

This questionnaire sheet has been put together to help let us know if the exercise program you did was acceptable to you.

1. How much did you enjoy the exercise program?

| 1 | 2 | 3 | 4 | 5 |
| --- | --- | --- | --- | --- |
| Did not  enjoy at all | Did not  enjoy much | Neutral | Enjoyed  a little | Enjoyed  very much |

# 2. How did you find doing three exercise sessions per week?

| 1 | 2 | 3 |
| --- | --- | --- |
| Not enough | Just right | Too many |

# 3. How did you find exercising for one hour each session?

| 1 | 2 | 3 |
| --- | --- | --- |
| Too short | Just right | Too long |

# 4. How did you find the difficulty of the sessions? (intensity)

| 1 | 2 | 3 |
| --- | --- | --- |
| Too easy | Just right | Too hard |

# 5. Do you think the exercise program helped your ability to do other exercise like sport or school PE?

| Yes | No |
| --- | --- |

# 6. What did you like about the exercise program?

__________________________________________________________________________________________________________________________

# 7. What didn’t you like about the exercise program?

__________________________________________________________________________________________________________________________

8. Would you participate in a similar exercise program in the future?

____Yes. Why:

_____________________________________________________________

___No. Why not:

_____________________________________________________________

# 9. Please provide any other comments or recommended changes to the exercise program:

_____________________________________________________________
